# MARU-MTL: A Mamba-Enhanced Multi-Task Learning Framework for Continuous Blood Pressure Estimation Using Radar Pulse Waves

**DOI:** 10.3390/bioengineering13030320

**Published:** 2026-03-11

**Authors:** Jinke Xie, Juhua Huang, Chongnan Xu, Hongtao Wan, Xuetao Zuo, Guanfang Dong

**Affiliations:** 1 School of Advanced Manufacturing, Nanchang University, Nanchang 330031, China; xjk98@email.ncu.edu.cn (J.X.); 412400230101@email.ncu.edu.cn (C.X.); worlft@email.ncu.edu.cn (H.W.); zxt91@email.ncu.edu.cn (X.Z.); 2 Department of Computing Science, University of Alberta, Edmonton, AB T6G 2R3, Canada; guanfang@ualberta.ca

**Keywords:** noncontact blood pressure estimation, radar, variational autoencoder-based signal quality index, multi-task learning, bidirectional Mamba

## Abstract

Continuous blood pressure (BP) monitoring is essential for the prevention and management of cardiovascular diseases. Traditional cuff-based methods cause discomfort during repeated measurements, and wearable sensors require direct skin contact, limiting their applicability. Radar-based contactless BP measurement has emerged as a promising alternative. However, radar pulse wave (RPW) signals are susceptible to motion artifacts, respiratory interference, and environmental clutter, posing persistent challenges to estimation accuracy and robustness. In this paper, we propose MARU-MTL, a Mamba-enhanced multi-task learning framework for continuous BP estimation using a single millimeter-wave radar sensor. To address signal quality degradation, a Variational Autoencoder-based Signal Quality Index (VAE-SQI) mechanism is proposed to automatically screen RPW segments without manual annotation. To capture long-range temporal dependencies across cardiac cycles, we integrate a Bidirectional Mamba module into the bottleneck of a U-Net backbone, enabling linear-time sequence modeling with respect to the segment length. We also introduce a multi-task learning strategy that couples BP regression with arterial blood pressure waveform reconstruction to strengthen physiological consistency. Extensive experiments on two datasets comprising 55 subjects demonstrate that MARU-MTL achieves mean absolute errors of 3.87 mmHg and 2.93 mmHg for systolic and diastolic BP, respectively, meeting commonly used AAMI error thresholds and achieving metrics comparable to BHS Grade A.

## 1. Introduction

Cardiovascular diseases remain the leading cause of mortality worldwide [1]. Hypertension, a primary risk factor for stroke, coronary heart disease, and heart failure [2], affects approximately 1.28 billion adults globally [3] and is often associated with complications such as renal impairment [4]. Therefore, accurate and convenient blood pressure measurement is essential for the prevention, diagnosis, and management of cardiovascular diseases.

BP measurement techniques can be broadly categorized into invasive and noninvasive methods. Invasive measurement via intra-arterial catheter is regarded as the clinical gold standard [5], but its procedural complexity and risks of complications restrict its use to intensive care settings [6]. Noninvasive measurement avoids invasive access to the body, and can be further divided into contact-based and noncontact approaches.

Contact-based methods are dominated by cuff-based devices, whose repeated inflation causes discomfort during frequent monitoring. Photoplethysmography (PPG)-based wearables enable continuous BP estimation through pulse transit time (PTT) or waveform morphology analysis [7]. However, PTT exhibits substantial inter-individual variability and requires subject-specific calibration [8]. Moreover, PPG signals are susceptible to motion artifacts, ambient temperature, and skin tone, and all contact-based methods require direct skin contact, limiting their applicability.

Radar has been increasingly explored as a promising modality for noncontact physiological sensing [9]. Millimeter-wave radar can penetrate clothing and extract pulse-wave information from sub-millimeter chest-surface micro-displacements induced by cardiac activity [10,11]. These advantages have driven increasing interest in radar-based approaches for noncontact and continuous blood pressure monitoring.

The morphology of the pulse wave encodes rich cardiovascular information and is tightly coupled to BP. Early radar-based studies estimated BP by deriving PTT and pulse wave velocity (PWV) from radar signals combined with auxiliary sensors. Buxi et al. combined radar with bioimpedance and ECG to compute PTT for central arterial BP estimation [12]. Ebrahim et al. fused radar, PPG, and ECG to extract temporal indices for continuous SBP estimation [9], while Lauteslager et al. used multi-site ultra-wideband (UWB) radar with ECG synchronization [13]. However, these methods rely on additional sensors and therefore do not enable single-sensor monitoring.

An increasing body of work has begun to investigate BP monitoring using a single radar sensor alone. These studies aim to simplify system deployment and improve user convenience by extracting BP-related features directly from radar pulse wave (RPW) signals, without requiring additional contact-based sensors.

Several studies have explored single-radar BP estimation through PTT-based approaches. Geng et al. derived central artery PTT (caPTT) from simultaneously acquired carotid and thoracic pulse signals [14]. Zhao et al. extracted carotid–femoral PTT (cf-PTT) from UWB radar measurements of the central aorta [15], while Tseng et al. estimated BP from wrist pulse waves using reflective PTT (R-PTT) [16]. Although these methods demonstrate the feasibility of hemodynamic modeling with a single radar, they typically require subject-specific baseline calibration, and their long-term reliability remains uncertain.

To eliminate the need for auxiliary sensors and subject-specific calibration, researchers have turned to deep learning for end-to-end BP regression from radar signals. Jiang et al. proposed a Transformer-based framework that encodes radar pulse wave sequences into global temporal representations for direct systolic blood pressure (SBP) and diastolic blood pressure (DBP) regression [17]. Qiu et al. introduced deformable convolution to enhance local morphological representations [18], while Zhao et al. employed a modified ResNet to learn pulse wave morphology–BP relationships [19]. Dual-stage architectures have also been explored to decouple feature extraction from regression [20]. Despite these advances, several challenges remain. First, most methods lack an unsupervised signal quality control mechanism, leaving them sensitive to corrupted inputs. Second, the scarcity of large-scale, high-quality RPW datasets with synchronized reference BP increases the risk of overfitting. Third, scalar BP regression alone provides limited supervision and does not enforce physiological consistency between RPW morphology and hemodynamics, limiting cross-scenario robustness.

To address the above challenges, an end-to-end learning framework is developed for single-radar-based contactless BP estimation. The proposed framework aims to stably extract RPW features associated with BP and to improve robustness under cross-scenario and long-term monitoring. Radar derived pulse waves are used as input, together with data augmentation and an unsupervised quality control mechanism. A multi-task deep network is employed to jointly learn scalar BP regression and arterial blood pressure (ABP) waveform representation, enabling BP estimation without additional contact-based sensors or subject-specific baseline calibration. The main contributions are summarized as follows.

(1) We propose an unsupervised signal-quality evaluation and screening strategy for RPW. Specifically, we develop a variational autoencoder-based signal quality index (VAE-SQI) that automatically identifies and filters out anomalous segments, providing unified quality control during both training and inference without manual thresholds or heuristic prescreening.

(2) We design a multi-task deep learning network, termed Mamba-enhanced Attention Residual U-Net with Multi-Task Learning (MARU-MTL), by integrating a bidirectional Mamba (Bi-Mamba) module into a residual U-Net backbone with multi-scale attention. This architecture efficiently captures long-range dependencies in high-sampling-rate radar sequences while preserving local waveform details through multi-channel derivative inputs.

(3) We develop a multi-task learning mechanism that couples scalar BP regression with ABP waveform reconstruction, strengthening physiological-consistency constraints and improving generalization across scenarios and subjects.

The remainder of this paper is organized as follows. Section 2 describes the proposed method. Section 3 presents the experimental setup and main results. Section 4 provides ablation studies and comparisons with existing methods, and discusses limitations. Section 5 concludes the paper.

## 2. Materials and Methods

**Figure 1:**
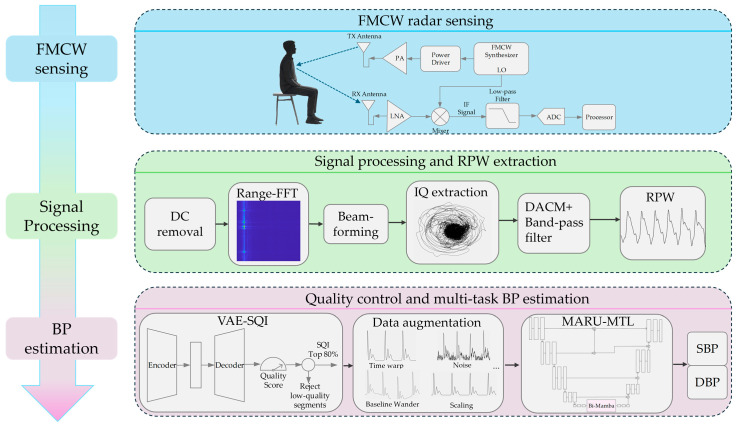
Overall flowchart.

This section outlines the proposed radar-based contactless BP estimation method and its key modules. The overall flowchart is shown in Figure 1. First, the Frequency-Modulated Continuous Wave (FMCW) radar principle and the RPW acquisition procedure are described. Next, the signal preprocessing pipeline for RPW is presented, together with data augmentation and a VAE-SQI screening mechanism to improve data validity and model generalization. Finally, the multi-task deep learning framework integrating Bi-Mamba and the associated loss function design are introduced, providing the methodological basis for subsequent experiments.

### 2.1. FMCW Radar Principle

FMCW radar probes targets by transmitting a linearly frequency-modulated chirp signal. The transmit signal can be expressed as

(1) $$s_{T}(t)=A_{T}\;\cos\left(2\pi f_{c}t+\pi \frac{B}{T_{c}}t^{2}+\varphi_{0}\right)$$

where *A_T_* denotes the transmit amplitude, *f_c_* is the carrier frequency, *B* is the modulation bandwidth, *T_c_* is the chirp duration, and *φ*_0_ is the initial phase. The backscattered echo is received, amplified by a low-noise amplifier (LNA), and mixed with the local oscillator to generate an intermediate frequency (IF) signal, which is then low-pass filtered and digitized by an analog-to-digital converter (ADC). After mixing, the IF signal contains both range and motion information. For chest micro-motion sensing, the target range can be decomposed as

(2) $$R\left(t\right)=R_{0}+x\left(t\right)$$

where *R*_0_ is the static range and *x*(*t*) represents the small displacement induced by respiration and cardiac activity.

### 2.2. Signal Processing

In practical measurement environments, the radar return contains not only the target echo from the human chest wall but also reflections from static objects, such as walls and furniture, as well as system-induced DC offsets. These interference components can severely degrade subsequent vital sign extraction.

To suppress static clutter, mean subtraction is applied along the slow time dimension. Let *S_IF_*(*m*, *n*) denote the intermediate frequency (IF) signal at the *n*-th sample of the *m*-th chirp. The clutter suppressed signal is given by

(3) $${s^{\prime }}_{IF}(m,n)=s_{IF}(m,n)-\frac{1}{M}\sum_{m=1}^{M}s_{IF}(m,n)$$

where *M* is the number of chirps. This operation removes the time-invariant component dominated by stationary reflectors.

DC offsets in the I/Q channels are estimated using a sliding-window mean and subtracted from the respective signals.

To enhance the SNR of chest micro-motion signatures, digital beamforming is employed. For an array with *N* receive elements, the complex baseband signal from the n-th channel is

(4) $$M_{n}(t)=A_{n}e^{j\varphi_{n}(t)}$$

where *A_n_* and *φ_n_*(*t*) denote the amplitude and phase of the received signal, respectively. The phase term *φ_n_*(*t*) includes both the propagation-induced phase component and the phase variations caused by target micro motion.

To steer the main lobe toward the chest direction *θ*, each channel is multiplied by a direction matched phase compensation weight *W_n_*(*θ*), determined by the array geometry. The weight is determined by the array geometry and can be expressed as:

(5) $$W_{n}(\theta )=e^{-j\frac{2\pi d(n-1)\sin(\theta )}{\lambda }}$$

where d is the inter-element spacing, *λ* is the carrier wavelength. The beamformed output for the four-channel array is

(6) $$BF(\theta ,t)=\sum_{n=1}^{4}W_{n}^{*}(\theta )M_{n}(t)$$

Phase stability across candidate range-angle combinations is evaluated to select the optimal configuration, significantly enhancing the SNR and spatial consistency of the output phase.

To extract phase information, the Extended Differentiate and Cross-Multiply (DACM) algorithm is employed to avoid the phase-wrapping artifacts of conventional arctangent demodulation. Its discrete form is

(7) $$\Phi [n]=\sum_{k=2}^{n}\frac{I[k]\{Q[k]-Q[k-1]\}-Q[k]\{I[k]-I[k-1]\}}{I^{2}[k]+Q^{2}[k]}$$

where *Φ*[*n*] denotes the demodulated phase at the *n*-th sample in radians, and *I*[*k*] and *Q*[*k*] are the in-phase and quadrature components after DC offset removal.

### 2.3. Unsupervised RPW Quality Assessment and Screening Based on VAE-SQI

**Figure 2:**
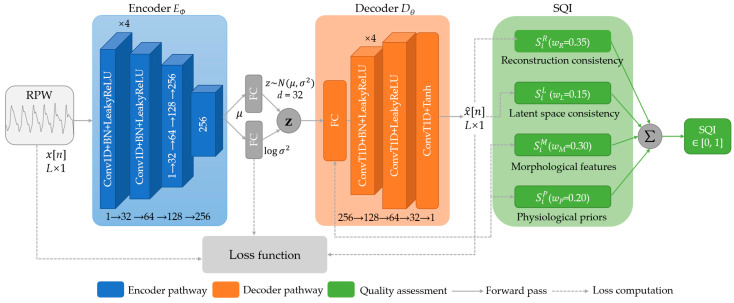
Overall process of VAE-SQI.

In practical measurements, RPW signals are often contaminated by motion artifacts, respiratory coupling, variations in scattering paths, and residual environmental clutter, which can lead to waveform distortion, weakened periodicity, or elevated high-frequency noise. Such low-quality segments not only dilute effective supervision but may also cause deep models to learn spurious correlations unrelated to blood pressure, thereby degrading generalization across scenarios and over long-term monitoring. Because segment-level manual annotation of RPW is costly and inherently subjective, a VAE-SQI-based unified unsupervised quality control module is introduced to quantify and screen RPW segments without relying on blood pressure labels. This module provides high-confidence inputs for subsequent data augmentation and multi-task learning. The overall process of VAE-SQI is shown in Figure 2.

The continuous RPW is segmented into N fixed-length windows ${\left\{x_{i}\right\}}_{i=1}^{N}$ with a window length *L*. An overlapped sliding-window scheme is adopted to increase sample coverage. To reduce the influence of inter-segment amplitude variations and slow trends on model training, each segment is standardized to zero mean and unit variance, yielding ${\tilde{x}}_{i}$, which is used as the input to the VAE. The normalized input ${\tilde{x}}_{i}$ is computed as

(8) $${\tilde{x}}_{i}=\frac{x_{i}-\mu (x_{i})}{\sigma (x_{i})+\varepsilon }$$

where *μ*(*x_i_*) and *σ*(*x_i_*) denote the mean and standard deviation of segment *x_i_*, respectively, and *ɛ* is a small constant to prevent division by zero.

To prevent VAE training from being dominated by a large number of anomalous segments, VAE-SQI introduces a lightweight pre-screening strategy. For each segment, a set of physiology- and statistics-consistency indicators reflecting periodicity is computed to obtain a pre-screening score $s_{i}^{p}$. This score jointly characterizes the periodicity and spectral plausibility of RPW, including autocorrelation-based periodicity strength, energy proportion within the heart-rate band, suppression of abrupt spikes, mitigation of baseline drift, regularity of inter-peak intervals, and the proportion of high-frequency noise. Subsequently, segments are ranked by $s_{i}^{p}$, and a fixed proportion of top-ranked segments is selected as a candidate high-quality subset for stable VAE training.

A one-dimensional variational autoencoder is trained on the prescreened candidate set to learn the latent distribution of high-quality RPW segments and to reconstruct the input waveform. The VAE encoder maps the normalized segment ${\tilde{x}}_{i}$ to the parameters of the latent Gaussian distribution (*μ_i_*, log*σ_i_*^2^). A latent variable *z_i_* is then sampled through the reparameterization trick, and the decoder reconstructs the corresponding waveform ${\tilde{x}}_{i}$. The training objective consists of a reconstruction term and a Kullback–Leibler (KL) divergence regularizer, which together encourage faithful waveform reconstruction while constraining the latent space to be continuous and well-structured [21]. Accordingly, the VAE loss is defined as

(9) $$L_{\mathrm{VAE}}=L_{\mathrm{rec}}+\beta D_{\mathrm{KL}}\left(q_{\phi }(z\;|\;{\tilde{x}}_{i})∥p(z)\right)$$

D_KL_ denotes the KL divergence between the variational posterior $q_{\phi }(z|\tilde{x})$ and the standard normal prior *p*(*z*), and *β* is a balancing coefficient that controls the strength of latent regularization. The reconstruction loss is computed as

(10) $$L_{\mathrm{rec}}=\frac{1}{L}{\left\|{\tilde{x}}_{i}-{\hat{x}}_{i}\right\|}_{2}^{2}$$

To improve training stability, we adopt a phased training strategy. First, we train the VAE on the prescreened subset until convergence, and then gradually incorporate the remaining segments for fine-tuning. This strategy expands the coverage of data distribution and retains the ability of the model to capture high-quality RPW.

After VAE training, a signal quality index is computed for each RPW segment by integrating multi-dimensional metrics. VAE-SQI evaluates segment quality from four perspectives: reconstruction consistency, latent space consistency, morphological features, and physiological priors. The definition and weight of each component are summarized in Table 1. The overall VAE-SQI score is computed as a weighted sum of the component scores. The weights are selected via validation-set tuning to balance reconstruction fidelity, latent-space regularity, and morphology/physiology plausibility, and are fixed for all experiments.

The four components are normalized and fused through weighted combination to obtain the final VAE-SQI score:

(11) $$S_{i}=w_{R}S_{i}^{R}+w_{L}S_{i}^{L}+w_{M}S_{i}^{M}+w_{P}S_{i}^{P}$$

**Figure 3:**
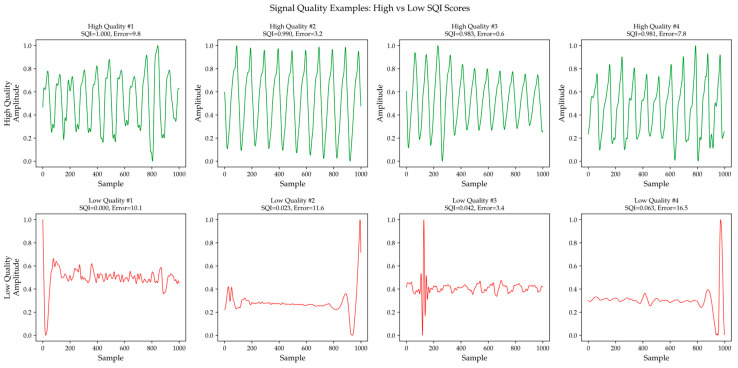
Examples of high-quality and low-quality segments.

Based on the statistical properties of the *S_i_* distribution, an adaptive threshold τ is employed to classify segments into high-quality and low-quality segments. Figure 3 presents typical waveform examples of high-quality and low-quality segments.

The VAE encoder consists of four convolutional stages with channel dimensions of 32, 64, 128, and 256, each using Conv1D with kernel size 7 and stride 2, followed by batch normalization, LeakyReLU at slope 0.2, and one residual block. The latent space has dimension 64. The decoder mirrors the encoder with transposed convolutions and a final Tanh activation. The VAE was trained using a two-stage strategy: Stage 1 trained for 100 epochs on pre-filtered data, specifically the top 35% ranked by the heuristic pre-screening score, at a learning rate of 3 × 10^−4^; Stage 2 fine-tuned for 60 epochs on the full dataset at a reduced learning rate of 1.5 × 10^−5^. The Kullback–Leibler (KL) weight β was linearly warmed up from 0.01 to 0.5 over 30 epochs.

**Figure 4:**
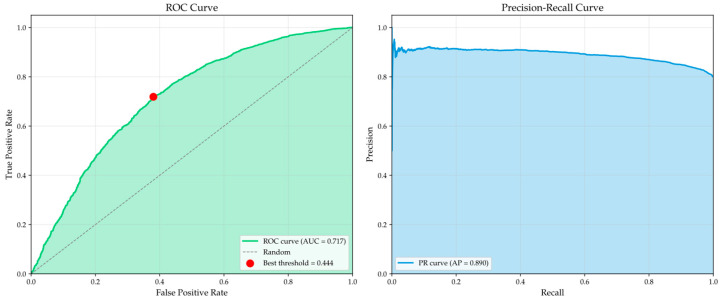
ROC curve and Precision–Recall curve for VAE-SQI quality assessment.

To validate the effectiveness of VAE-SQI, a quantitative evaluation was conducted using manually annotated quality labels. As shown in Figure 4 and Table 2, VAE-SQI achieved an average precision (AP) of 0.890 and an F1-score of 0.864, indicating that the segments identified as high-quality are predominantly genuine, with reliable precision–recall trade-offs. The Receiver Operating Characteristic Area Under Curve (ROC-AUC) of 0.717 is relatively lower, partly because the manually annotated binary labels impose a hard boundary on what is inherently a continuous quality spectrum, and segments near the boundary are intrinsically ambiguous. Additionally, RPW signals exhibit substantial inter-individual morphological variability due to differences in arterial stiffness, chest wall geometry, and cardiovascular dynamics. A segment appearing atypical relative to the population average may still carry valid physiological information for a specific individual. As a continuous scoring mechanism rather than a binary classifier, VAE-SQI is designed to accommodate this diversity by learning a broad latent distribution of plausible RPW patterns, and its effectiveness is more appropriately evaluated by its downstream impact on BP estimation. Correlation analysis between the VAE-SQI score and BP estimation error yielded Pearson *r* = −0.316 and Spearman ρ = −0.291. The moderate correlation is expected, since estimation error depends on multiple factors beyond signal quality, including physiological state difficulty and inter-subject variability.

### 2.4. Data Augmentation

In radar-based blood pressure monitoring scenarios, the acquisition of high-quality RPW data is costly, and the coverage of subjects and physiological states is limited. To mitigate the overfitting risk caused by data scarcity and improve model generalization, this study introduces multiple data augmentation strategies that apply reasonable transformations to RPW signals while preserving blood pressure labels, simulating signal variations that may occur in practical monitoring.

Five augmentation operations are designed to account for the temporal variability and physiological characteristics of RPW signals, including time shift, amplitude scaling, Gaussian noise injection, time warping, and baseline wander. Time shift performs circular shifting of the waveform to emulate random offsets in the detected cardiac onset, thereby improving robustness to phase misalignment. Amplitude scaling randomly adjusts signal magnitude within a predefined range to model inter-subject variations in backscattered intensity. Gaussian noise injection adds stochastic perturbations to the waveform to enhance tolerance to measurement noise. Time warping applies mild temporal stretching or compression via interpolation to mimic cycle-to-cycle variability induced by physiological fluctuations. Baseline wander introduces a low-frequency drift component to simulate slow baseline variations caused by respiration and other physiological factors.

Specifically, an augmentation factor of 3× was used, meaning each original segment generates three augmented copies. Each augmentation type was applied independently with a probability of 0.5. The time shift range was set to ±10% of the window length, amplitude scaling gain was sampled uniformly between 0.9 and 1.1, additive Gaussian noise level was *σ_g_* = 0.02 relative to the signal standard deviation, time warping ratio ranged between 0.95 and 1.05, and baseline wander was implemented as a sinusoidal drift with amplitude 0.05 and frequency 0.5 Hz.

### 2.5. Blood Pressure Estimation Model

The U-Net architecture, initially proposed for biomedical image segmentation, employs a symmetrical encoder–decoder structure that retains fine-grained multi-scale waveform features through skip connections [22]. This design is highly suitable for physiological signal processing, where both global context and local waveform details are crucial for accurate blood pressure estimation.

**Figure 5:**
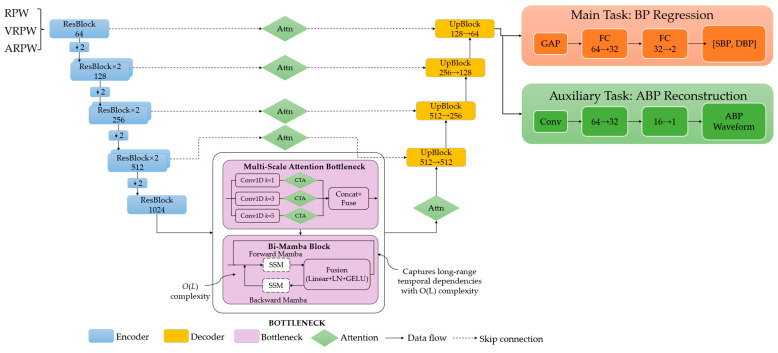
Architecture of MARU-MTL.

To accurately estimate blood pressure from RPW signals, we propose MARU-MTL, illustrated in Figure 5, a deep learning architecture specifically designed for continuous BP estimation. The proposed architecture consists of a U-Net encoder–decoder backbone with attention-guided skip connections for hierarchical feature extraction, a Bi-Mamba bottleneck for temporal sequence modeling, and a multi-task learning framework that jointly performs BP regression and ABP waveform reconstruction. The network components are described in detail as follows.

As illustrated in Figure 5, MARU-MTL adopts an encoder–decoder architecture built upon a residual U-Net backbone. The encoder is composed of stacked residual blocks (ResBlocks) with downsampling, while the decoder uses upsampling blocks (UpBlocks) to progressively recover temporal resolution. Attention gates (Attn) are applied on skip connections to suppress irrelevant activations and emphasize BP-related features. At the bottleneck, we employ a multi-scale attention module to aggregate features at different receptive fields, followed by a bidirectional Mamba (Bi-Mamba) block implemented with state space models (SSMs) to capture long-range temporal dependencies efficiently. The overall network branches into two heads: a main regression head that predicts SBP/DBP using global average pooling (GAP) and fully connected (FC) layers, and an auxiliary reconstruction head that outputs the ABP waveform via convolutional layers.

#### 2.5.1. Encoder

The input to MARU-MTL comprises three-channel physiological signals derived from the RPW. The first channel is the RPW, while the second and third channels correspond to its first-order derivative, referred to as the velocity RPW (VRPW), and its second-order derivative, referred to as the acceleration RPW (ARPW), respectively. VRPW and ARPW are incorporated based on their established associations with arterial stiffness and BP variability. These derivative signals enhance the representation of waveform morphology, particularly features closely related to BP dynamics, such as the systolic upstroke slope and the dicrotic notch. The three-channel input is denoted as X ∈ *R*^(3×*L*)^, where *L* is the segment length at a sampling rate of 100 Hz. To ensure consistent feature scaling across channels, we apply L2 normalization to the input.

The encoder extracts hierarchical temporal features through five stages with progressively increased channel dimensions. Residual blocks are adopted as the core building unit of the encoder. This design facilitates improved gradient propagation, which is critical for training deep neural networks [23]. The basic residual block can be formulated as follows.

(12) $$y=F\left(x,\{W_{i}\}\right)+x$$

where x and y denote the input and output feature vectors of a layer, respectively. $F\left(x,\left\{W_{i}\right\}\right)$ represents the learned residual mapping, which is implemented by two consecutive one-dimensional convolutional layers followed by batch normalization (BN) and ReLU activation.

(13) $$F(x)=W_{2}\cdot \mathrm{BN}\left(\sigma \left(\mathrm{BN}(W_{1}\cdot x)\right)\right)$$

where σ(.) denotes the ReLU activation function. When the input and output dimensions differ, a 1 × 1 projection convolution is applied on the shortcut path to match feature dimensions. Each encoder stage consists of one or two residual blocks followed by a strided convolution for downsampling.

#### 2.5.2. Multi-Scale Attention Bottleneck

**Figure 6:**
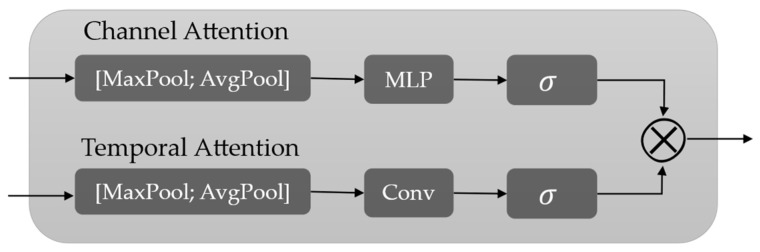
The CTA module structure.

To capture informative patterns at multiple receptive fields, a multi-scale attention mechanism is introduced at the bottleneck. Specifically, three parallel convolutional branches with kernel sizes 1, 3, and 5 are applied. For the *k*-th branch, the attention-enhanced feature is computed as

(14) $$Z_{k}=\mathrm{CTA}\left(\sigma \left(\mathrm{BN}\left({\mathrm{Conv}}_{k}(F)\right)\right)\right),\;k\in \{1,3,5\}$$

where **F** denotes the bottleneck input feature, Conv*_k_*(.) represents a 1D convolution with kernel size *k*, and CTA(.) denotes the channel–temporal attention module. The CTA module structure is shown in Figure 6.

The outputs of the three branches are then concatenated along the channel dimension and fused using a 1 × 1 convolution to obtain the multi-scale bottleneck representation. This fusion is formulated as

(15) $$F_{ms}=\sigma \left(\mathrm{BN}\left({\mathrm{Conv}}_{1\times 1}\left(\left[Z_{1};Z_{3};Z_{5}\right]\right)\right)\right)$$

where $\left[\cdot \;;\;\cdot \right]$ denotes channel-wise concatenation and Conv1 × 1(.) denotes a 1D pointwise convolution for feature fusion.

The CTA module adaptively recalibrates features via channel-wise and temporal attention. Given an input feature **F**, the channel attention map **M*_c_*** is computed by aggregating global descriptors using average pooling and max pooling, which are then passed through a shared multilayer perceptron (MLP). The channel attention is defined as

(16) $$M_{c}=\sigma \left(\mathrm{MLP}(\mathrm{AvgPool}(F))+\mathrm{MLP}(\mathrm{MaxPool}(F))\right)$$

where AvgPool(.) and MaxPool(.) denote global pooling operations, MLP(.) is a lightweight feed-forward network.

The temporal attention map **M*_t_*** is computed by pooling **F** along the channel dimension and applying a convolutional operator to capture local dependencies across time. The temporal attention is computed as

(17) $$M_{t}=\sigma \left(\mathrm{Conv}\left(\left[\mathrm{AvgPool}(F);\mathrm{MaxPool}(F)\right]\right)\right)$$

The final attention-refined feature is obtained by

(18) $$F^{\prime }=F⊙M_{c}⊙M_{t}$$

where ⊙ denotes element-wise multiplication with appropriate broadcasting.

#### 2.5.3. Bidirectional Mamba Block

Although convolutional layers are effective in capturing local patterns, BP estimation requires modeling dependencies spanning multiple cardiac cycles across the entire signal sequence. Mamba, a selective state-space (SSM) model-based architecture, provides an efficient Transformer alternative for sequence modeling, with linear computational complexity *O*(*L*) rather than the quadratic cost *O*(*L*^2^) [24]. The core SSM is defined by the following continuous-time state equations.

(19) $$h^{\prime }(t)=Ah(t)+Bx(t),\;\;y(t)=Ch(t)$$

where $A\in R^{N\times N}$ denotes the state transition matrix, $B\in R^{N\times 1}$ and $C\in R^{1\times N}$ are projection matrices, and *N* is the state dimension. Here, h(t), x(t), and $y\left(t\right)$ represent the hidden state, input, and output at time *t*, respectively. The Mamba layer instantiates a selective SSM that performs content-aware state updates, thereby facilitating effective long-range contextual modeling.

**Figure 7:**
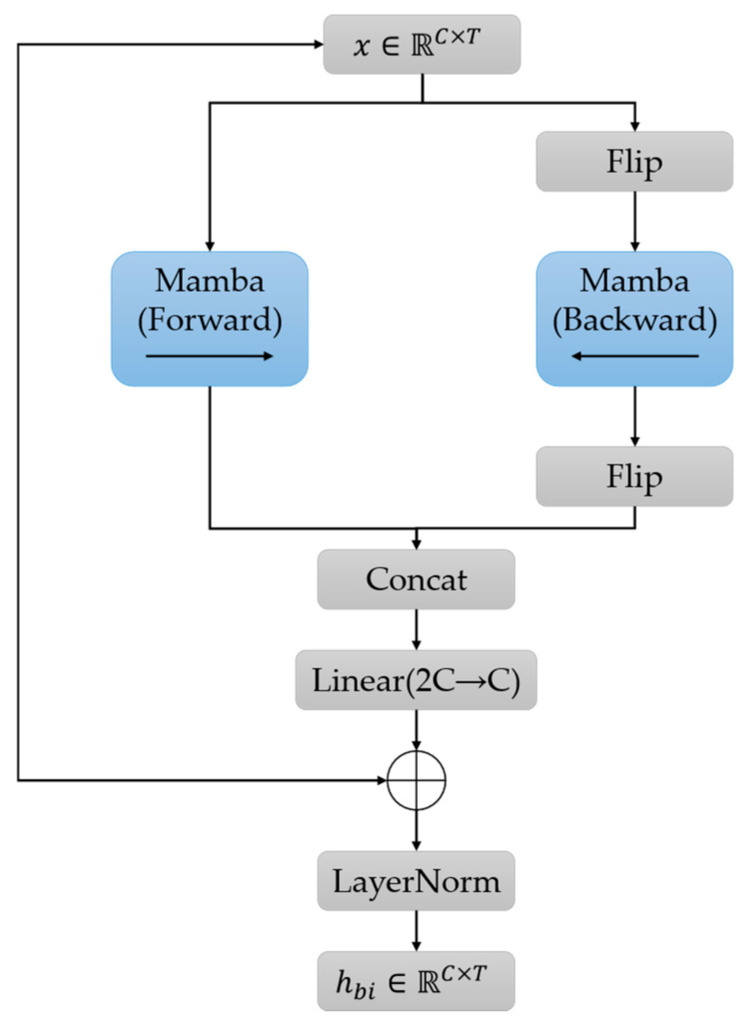
Schematic of the proposed Bi-Mamba architecture. A forward branch and a backward branch are applied to the input sequence, and their outputs are concatenated and then fused through a linear projection layer.

The standard Mamba module operates in a causal manner and thus can only exploit information from preceding time steps. In this work, BP estimation is formulated as an offline inference problem, where the complete signal window is accessible during prediction. Leveraging information from subsequent time steps can provide additional contextual cues and improve the stability of state estimation. Motivated by this observation, we develop a Bi-Mamba module, as depicted in Figure 7.

As illustrated in Figure 7, the input feature sequence **X** is processed by two independent Mamba branches in opposite directions. The forward branch captures dependencies from past to future, while the backward branch captures dependencies from future to past. This is formulated as

(20) $$h^{f}=\mathrm{Mamba}(F_{ms}),\;\;h^{b}=\mathrm{flip}(\mathrm{Mamba}(\mathrm{flip}(F_{ms})))$$

where **F**_ms_ denotes the multi-scale bottleneck feature, flip(.) reverses the sequence along the temporal dimension, and $h^{f}$ and $h^{b}$ are the forward and backward hidden representations, respectively. The representations from both directions are subsequently concatenated and fused via a linear projection equipped with a Gaussian error linear unit (GELU) activation.

Finally, a residual connection and Layer Normalization (LN) are applied to stabilize the training:

(21) $$h_{\mathrm{out}}=\mathrm{LN}\left(\mathrm{GELU}\left(\mathrm{Linear}\left(\left[h^{f};h^{b}\right]\right)+F_{ms}\right)\right)$$

This bidirectional design enables each temporal position in the bottleneck feature map to incorporate global context from the entire pulse-wave sequence.

#### 2.5.4. Decoder and Attention-Guided Skip Connections

The decoder mirrors the encoder structure with four upsampling stages, each consisting of upsampling via linear interpolation followed by a residual block, progressively restoring temporal resolution while reducing channel dimensions.

To refine feature fusion across skip pathways, we introduce attention-guided skip connections. The encoder features at each skip pathway are refined using the CTA module, which selectively emphasizes physiologically relevant components while suppressing noise. These refined features are concatenated with the upsampled decoder features, followed by a residual block for further optimization. This mechanism replaces the plain concatenation in standard U-Net and ensures that the decoder prioritizes hemodynamically informative patterns.

#### 2.5.5. Multi-Task Prediction Heads

The network employs two task-specific prediction heads to jointly perform BP regression and ABP waveform reconstruction, sharing the same encoder–decoder backbone.

The primary head predicts SBP and DBP from the final decoder features. Global average pooling (GAP) is first applied along the temporal dimension, followed by two fully connected (FC) layers with a nonlinear activation to obtain ${\hat{y}}_{SBP}$ and ${\hat{y}}_{DBP}$:

(22) $$\left[{\hat{y}}_{\mathrm{SBP}},{\hat{y}}_{\mathrm{DBP}}\right]={\mathrm{FC}}_{2}\left(\psi \left({\mathrm{FC}}_{1}\left(\mathrm{GAP}(d_{1})\right)\right)\right)$$

where **d**_1_ denotes the output of the last decoder stage, *ψ* denotes the ReLU activation function.

The auxiliary head reconstructs the continuous ABP waveform from the shared decoder features. It is implemented using two temporal convolutional layers followed by a 1×1 convolution for channel projection, progressively reducing the channel dimension and producing a single-channel waveform:

(23) $${\hat{y}}_{\mathrm{ABP}}={\mathrm{Conv}}_{3\times 3}\left(\mathrm{Conv}\left(\mathrm{Conv}(d_{1})\right)\right)\in {\mathbb{R}}^{1\times L}$$

### 2.6. Loss Function

The training objective is formulated as a multi-task loss, which jointly optimizes BP regression and ABP waveform reconstruction. Accordingly, the overall loss is defined as follows:

(24) $$L=L_{BP}+\lambda_{ABP}\cdot L_{ABP}$$

where *L_BP_* denotes the blood pressure regression loss, *L_ABP_* represents the ABP waveform reconstruction loss, and *λ_ABP_* is a weighting coefficient that balances the contribution of the auxiliary task.

For BP regression, the Huber loss is adopted for its robustness to outliers while maintaining sensitivity to small errors. The loss function is defined as

(25) $$L_{BP}=\lambda_{SBP}L_{\delta }\left({\hat{y}}_{SBP},y_{SBP}\right)+L_{\delta }\left({\hat{y}}_{DBP},y_{DBP}\right)$$

where $\hat{y}$ and y represent the predicted BP values and ground truth, *λ_SBP_* adjusts the weight of the systolic component, *δ* is the threshold for switching between quadratic and linear loss.

The Huber loss is formulated as:

(26) $$L_{\delta }(\hat{y},y)=\left\{\begin{array}{ll} \frac{1}{2}{\left(\hat{y}-y\right)}^{2}, & \mathrm{if}\;|\hat{y}-y|\leq \delta \\ \delta \left(|\hat{y}-y|-\frac{1}{2}\delta \right), & \mathrm{otherwise} \end{array}\right.$$

This hybrid loss stabilizes the optimization process and reduces sensitivity to outliers, thereby improving robust prediction.

The auxiliary reconstruction task aims to encourage the shared encoder to capture morphological features with hemodynamic significance. Instance Normalization (IN) is applied to both the predicted waveform and the true waveform before calculating the loss function. The reconstruction loss is calculated using the Smooth L1 loss function, as shown in the following formula.

(27) $$L_{ABP}=SmoothL1\left(\mathrm{Norm}\left({\hat{y}}_{ABP}\right),\mathrm{Norm}\left(y_{ABP}\right)\right)$$

where Norm(·) denotes instance normalization applied independently to each sample. The instance normalization eliminates inter-subject amplitude variations, allowing the reconstruction loss to focus on waveform morphology rather than absolute BP values.

The auxiliary ABP waveform reconstruction task is introduced for two main reasons. First, a continuous ABP waveform conveys substantially richer hemodynamic information than scalar BP values, including the systolic upstroke slope, the depth of the dicrotic notch, and the diastolic decay pattern. By constraining the network to reconstruct these morphological characteristics, the encoder is encouraged to learn a more comprehensive cardiovascular representation. Second, the shared encoder–decoder architecture provides implicit regularization through the auxiliary task, thereby reducing the risk of overfitting on a limited labeled dataset.

## 3. Experiments and Results

### 3.1. Dataset Description

This study conducts experiments on two synchronized Radar–ABP datasets. The first is a publicly collected dataset from 30 subjects covering five experimental conditions [25], and the second is an in-house dataset from 25 subjects including three conditions. In both datasets, an FMCW radar is used to capture chest micro-vibration signals, while ABP recorded by a continuous noninvasive blood pressure monitor serves as the ground truth.

The public dataset comprises 14 male subjects and 16 female subjects. The age ranges from 21 to 61 years, with a mean ± SD: 30.7 ± 9.9 years. For each subject, data were collected under five experimental conditions, including resting, Valsalva, apnea, tilt-up, and tilt-down. These conditions are designed to capture hemodynamic variations driven by different physiological regulations, thereby improving the model robustness to blood pressure fluctuations in practical scenarios.

**Figure 8:**
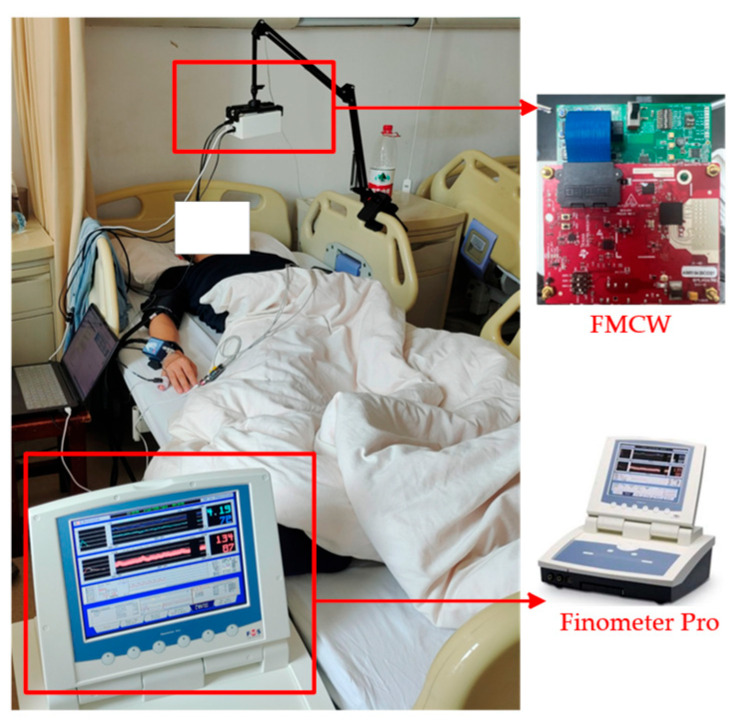
Experimental equipment and measurement scenario.

The in-house experiments employed an AWR1843 FMCW radar with a DCA1000 acquisition board, and a Finapres Medical Systems was used as the reference ABP monitor. The radar parameters are listed in Table 3, and the experimental setup is shown in Figure 8.

The in-house dataset includes 25 subjects (16 males, 9 females; mean ± SD: 46.6 ± 15.6 years), comprising 13 normotensive and 12 hypertensive individuals. Data were collected under three conditions (resting, Valsalva, apnea) in a controlled clinical environment at approximately 25 °C. The radar was positioned approximately 60 cm from the subject’s chest, with each session lasting 5 min. The two datasets are complementary in demographic composition, the public dataset is predominantly younger and normotensive, while the in-house dataset includes older subjects and hypertensive individuals. Together, the combined dataset spans an age range of 21–75 years, includes both sexes, and covers both normotensive and hypertensive populations across five distinct physiological conditions.

### 3.2. Experimental Setup

To reliably evaluate the model performance across different physiological conditions, the dataset is randomly split by subject into training, validation, and test sets with a ratio of 70%, 10%, and 20%, respectively. This protocol follows commonly adopted practices in data-driven blood pressure estimation studies and aims to maintain a balanced distribution of samples from different respiratory/postural conditions across the three subsets.

The MARU-MTL encoder follows a U-Net-style contracting path with five stages, containing 1, 2, 2, 2, and 1 residual blocks per stage respectively, with channel dimensions progressively increasing from 3 to 64, 128, 256, 512, and 1024. Each residual block consists of two Conv1D–BN–ReLU layers with kernel size 3 and a 1 × 1 shortcut projection when channel dimensions change. The bottleneck comprises a multi-scale attention module with three parallel branches at kernel sizes 1, 3, and 5, each followed by CTA attention and fused via 1 × 1 convolution, followed by a Bi-Mamba block with *d*_model_ = 1024, *d*_state_ = 16, *d*_conv_ = 4, and expand = 2, and a dropout layer at rate 0.1. The decoder mirrors the encoder with four UpBlocks, each using bilinear interpolation, CTA-gated skip connections with reduction ratio r = 4 and spatial kernel size 7, and one residual block. The BP regression head applies Global Average Pooling followed by two fully connected layers mapping from 64 to 32 to 2, with ReLU activation and dropout at rate 0.1. The ABP reconstruction head consists of three Conv1D layers reducing channels from 64 to 32 to 16 to 1, followed by interpolation to the target length.

The proposed MARU-MTL framework was implemented in PyTorch 2.9.1 and trained on an NVIDIA GeForce RTX 4090 GPU. AdamW was used for optimization with a weight decay of 1 × 10^−5^ and an initial learning rate of 1 × 10^−3^. A cosine annealing scheduler was applied to adapt the learning rate and facilitate stable convergence. The batch size was set to 512 and the model was trained for up to 120 epochs. To prevent overfitting, early stopping was employed, and training was terminated if the validation loss did not improve for 20 consecutive epochs.

For the multi-task loss, λ_SBP_ was set to 2.0 to account for the larger numerical range of SBP, while λ_ABP_ was set to 0.01 to balance the auxiliary ABP reconstruction task. The Huber loss threshold *δ* was set to 1 mmHg. During training, data augmentation was applied with an augmentation factor of 3×, meaning each original segment generates three augmented copies. Six augmentation types were applied independently, each with a probability of 0.5: time shift via circular shift up to ±10% of the window length, amplitude scaling with uniform random gain between 0.9 and 1.1, additive Gaussian noise at *σ_g_* = 0.02 relative to the signal standard deviation, time warping with a stretch/compress ratio between 0.95 and 1.05, baseline wander as a sinusoidal drift with amplitude 0.05 and frequency 0.5 Hz.

The VAE-SQI module was trained exclusively on segments from training-set subjects; no validation-set or test-set data were involved at any stage. After training, the VAE-SQI parameters were frozen and applied to all three splits in a forward-pass-only manner to compute segment-level quality scores. The top 80% of segments ranked by quality score were retained in each split, and the remaining 20% were excluded from both training and evaluation, as these segments predominantly exhibit severe waveform corruption such as loss of periodicity or dominant respiratory interference. The quality scores of the retained segments were further used as sample weights during BP model training to down-weight borderline-quality samples and reduce their gradient contributions.

The estimation accuracy of the proposed method is evaluated against the error criteria specified by the AAMI standard and the BHS grading protocol [26,27]. We conducted a comparative analysis between the proposed method and existing related methods. The following indicators are used to evaluate performance: mean error (ME), standard deviation (SD), mean absolute error (MAE), and root mean square error (RMSE).

### 3.3. Main Results

This section introduces the blood pressure estimation performance of the proposed MARU-MTL framework, encompassing overall performance metrics and performance analysis under various physiological states.

Table 4 summarizes the overall blood pressure estimation performance of MARU-MTL on the test set. MARU-MTL achieves a MAE of 3.87 mmHg and a SD of 5.90 mmHg for SBP estimation, and a MAE of 2.93 mmHg and a SD of 4.61 mmHg for DBP estimation. The Pearson correlation coefficients for SBP and DBP are 0.944 and 0.937, respectively, indicating a strong linear relationship between the predicted values and the reference blood pressure values. These results demonstrate the effectiveness of the proposed multi-task learning architecture in capturing the complex mapping from radar pulse waves to blood pressure dynamics.

Table 5 summarizes the per-dataset estimation performance, detailing the results for each cohort individually. The moderately higher errors observed in the in-house cohort are attributable to its broader age range and the inclusion of 12 hypertensive subjects. Nevertheless, both datasets individually achieve errors within the commonly referenced AAMI error thresholds, confirming the absence of dataset-specific systematic bias.

To assess whether the aggregate Pearson r is inflated by inter-subject BP variability, a within-subject analysis was conducted by computing MAE and r independently for each test subject. Per-subject MAE averaged 3.89 ± 1.32 mmHg for SBP and 2.99 ± 0.98 mmHg for DBP, closely matching the aggregate MAE. Per-subject *r* averaged 0.619 ± 0.259 for SBP and 0.563 ± 0.339 for DBP, considerably lower than the aggregate values. This discrepancy is expected because the intra-individual BP range during a single session is substantially narrower than the population-level range, limiting the achievable within-subject correlation. The consistency between per-subject and aggregate MAE confirms that the estimation accuracy is not an artifact of inter-subject variability, and that MAE provides a more reliable measure of per-subject tracking performance than aggregate r alone.

**Figure 9:**
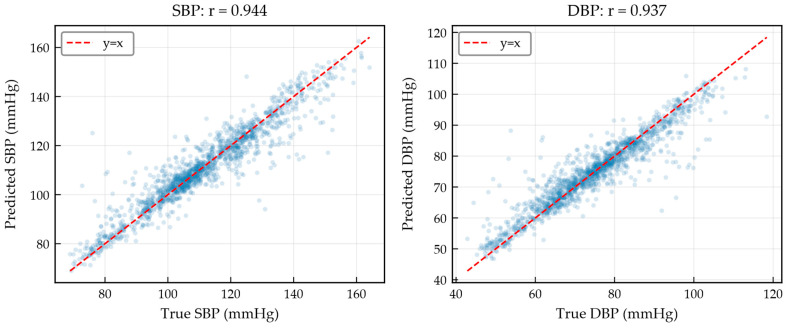
Scatter plots of SBP and DBP with linear regression analysis.

Figure 9 presents the scatter plots of predicted versus reference BP values for SBP and DBP with linear regression analysis. The data points are closely distributed along the identity line, with Pearson correlation coefficients of 0.944 and 0.937 for SBP and DBP, respectively. The tight clustering indicates strong agreement between predicted and reference values across the full physiological range. Notably, the estimation remains consistent at both low and high BP regions without evident proportional bias, suggesting that MARU-MTL does not systematically overestimate or underestimate blood pressure at the extremes of the distribution.

**Figure 10:**
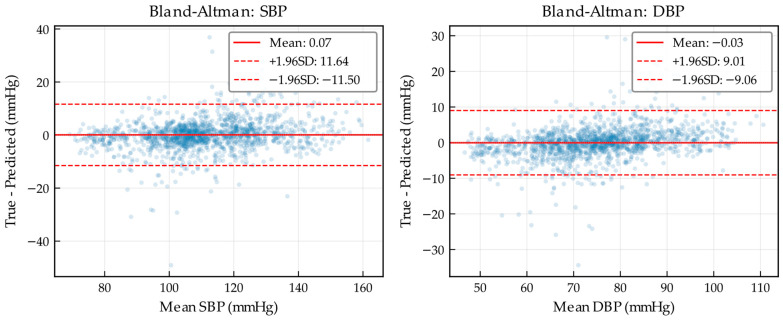
Bland–Altman diagram of SBP and DBP.

Figure 10 presents the Bland–Altman plots for SBP and DBP estimation. The errors are distributed around the zero line, indicating that MARU-MTL introduces negligible systematic bias overall. This stability is plausibly associated with the Bi-Mamba module embedded in the bottleneck, which models global temporal dependencies over the entire pulse sequence and enables prediction calibration according to the overall hemodynamic trend. In contrast to approaches dominated by local convolutional receptive fields that may be more susceptible to transient artifacts, the global modeling capability of Bi-Mamba can help reduce shifts induced by local aberrations.

Notably, the DBP error distribution is more compact, reflecting higher estimation precision and indirectly supporting the effectiveness of the multi-task learning strategy. The auxiliary ABP reconstruction task encourages the shared encoder to learn fine-grained morphological representations of the pulse waveform, which are closely related to accurate diastolic estimation. The scatter points are densely clustered around the mean line, without evident proportional bias or nonlinear patterns, suggesting that the estimation performance remains consistent across the physiological range. In addition, this behavior is consistent with the use of the Huber loss, which attenuates the influence of hard or outlier samples on gradient updates and thereby reduces the risk of overfitting to noisy radar segments.

**Figure 11:**
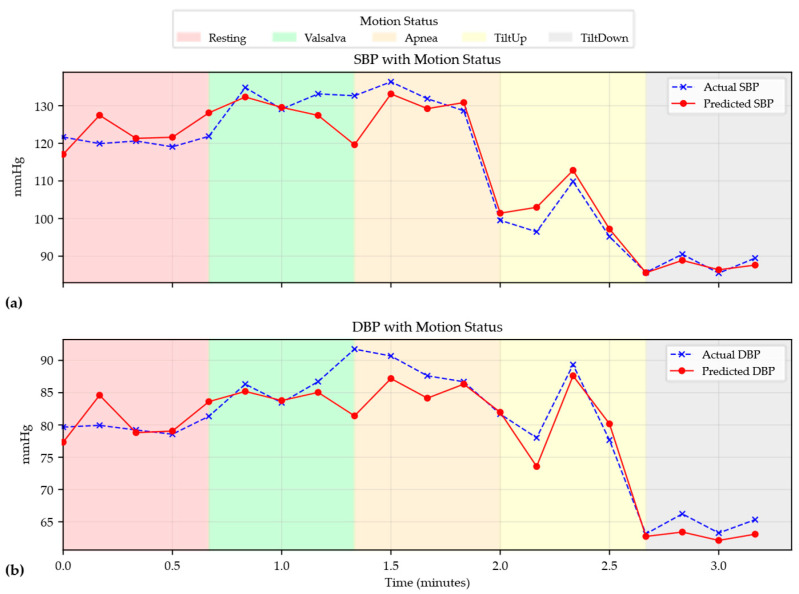
(**a**) Continuous tracking of SBP for a representative subject transitioning through multiple physiological states. (**b**) Continuous tracking of DBP for a representative subject transitioning through multiple physiological states.

To evaluate the capability of the model to capture dynamic hemodynamic variations in real time, a continuous tracking experiment was conducted on subjects undergoing multiple physiological state transitions, as illustrated in Figure 11. Across the entire time series, the predicted blood pressure closely follows the reference measurements, demonstrating a high level of temporal consistency.

**Figure 12:**
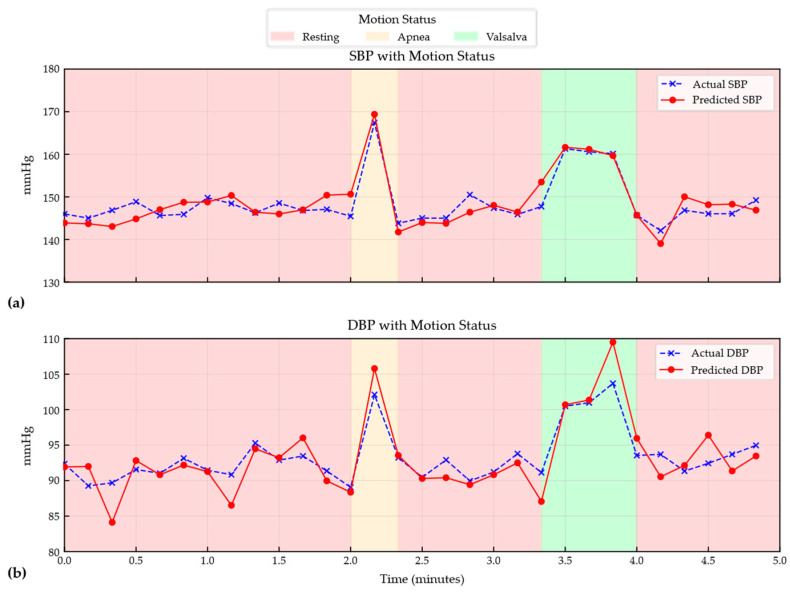
(**a**) Continuous tracking of SBP for an in-house dataset representative subject transitioning through multiple physiological states. (**b**) Continuous tracking of DBP for an in-house dataset representative subject transitioning through multiple physiological states.

Figure 12 illustrates the continuous blood pressure tracking results for a representative subject from the in-house dataset during transitions across multiple physiological states. The predicted curves exhibit a high degree of agreement with the reference measurements throughout the time series and effectively track the dynamic variations in blood pressure, indicating that the proposed method maintains robust dynamic responsiveness and temporal consistency on self-collected data as well.

MARU-MTL shows strong sensitivity across different physiological mechanisms. During the initial resting phase, high accuracy is achieved under relatively stable conditions. Importantly, during apnea, the model continues to track subtle blood pressure fluctuations associated with changes in intrathoracic pressure and hypoxia-induced autonomic responses. During the Valsalva maneuver, the characteristic rapid rise in blood pressure and the subsequent recovery are captured with no evident phase lag. Similarly, during the tilt-up and tilt-down tests, the system adapts to posture-induced hemodynamic shifts. This temporal fidelity is plausibly attributable to the Bi-Mamba bottleneck, which facilitates modeling long-range dependencies and global temporal context within the RPW sequence. By integrating information across time steps within each window, the model can better distinguish genuine physiological changes from transient artifacts, enabling stable tracking under challenging conditions.

**Figure 13:**
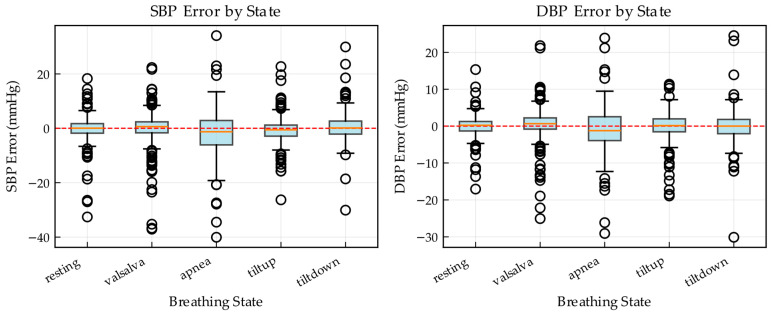
Statistical distribution of prediction errors for SBP and DBP grouped by physiological states.

To further quantify the generalization performance under different hemodynamic conditions, Figure 13 presents the distribution of prediction errors grouped by experimental condition. Resting serves as a key performance baseline and exhibits the narrowest interquartile range (IQR) with the tightest clustering of errors around zero, confirming that MARU-MTL achieves high-precision estimation under near-artifact-free conditions. Notably, the model remains highly robust during apnea, with an error variance comparable to the resting baseline and markedly lower than that observed in the dynamic tilt conditions, suggesting effective tracking of hemodynamic fluctuations induced by respiration-related regulation.

In more dynamic scenarios, such as the Valsalva and tilt-up, the error variance increases slightly, likely due to rapid cardiovascular adjustments and motion-related artifacts. Across all five conditions, the median errors for both SBP and DBP remain close to 0 mmHg, indicating the absence of condition-specific systematic bias. Overall, these results suggest that the multi-scale attention bottleneck extracts robust morphological features that persist even under signal distortions, enabling the decoder to reconstruct accurate blood pressure values across varying activity states.

**Figure 14:**
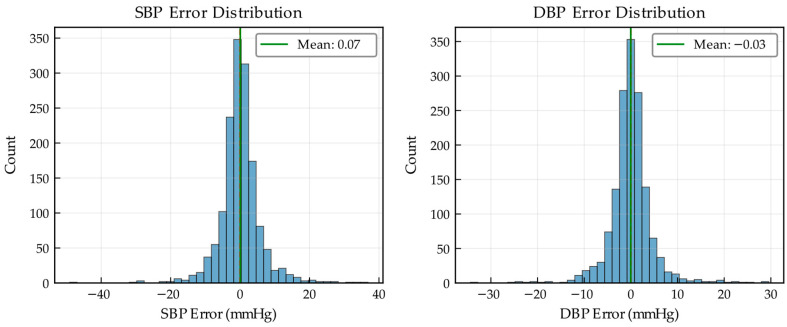
Histogram and kernel density estimate of prediction errors for SBP and DBP.

The statistical characteristics of the prediction errors are examined using the histograms and kernel density estimates shown in Figure 14.

For both SBP and DBP, the error distributions exhibit an approximately zero-centered Gaussian shape with pronounced kurtosis and near-symmetry, confirming the absence of systematic bias and indicating that the majority of predictions incur only minor errors across all physiological conditions.

To evaluate the computational efficiency of the proposed model, inference time was measured on GPU. MARU-MTL contains 33.22 M parameters and requires 4.62 GFLOPs per forward pass. The single-sample mean inference time is 4.68 ms. In batch mode, the model achieves a peak throughput of 1737 samples/s at batch size 32. These results confirm that the computational cost of MARU-MTL is negligible relative to the data acquisition window, fully satisfying the requirements for real-time continuous blood pressure monitoring.

## 4. Discussion

The experimental results presented in Section 3 demonstrate that the method achieves competitive blood pressure estimation accuracy across multiple physiological conditions. To further contextualize these findings, this section first benchmarks MARU-MTL against state-of-the-art radar-based BP estimation methods, followed by systematic ablation studies that quantify the individual contributions of each proposed component. Finally, the limitations of the current study and directions for future improvement are discussed.

### 4.1. Comparison with State-of-the-Art Methods

To rigorously assess the standing of MARU-MTL, the proposed method was comprehensively benchmarked against a range of state-of-the-art radar-based blood pressure estimation approaches published in recent years. The comparison covers different measurement sites and model architectures. The quantitative results are summarized in Table 6. The proposed MARU-MTL achieves strong performance across all evaluation metrics, indicating superior accuracy and precision compared with the competing approaches.

Compared with prior approaches, MARU-MTL demonstrates several advantages. Unlike CNN + LSTM [28], the proposed method incorporates hypertensive participants and achieves substantially lower estimation errors through effective temporal modeling. Unlike TRCCBP [17], the framework supports continuous BP tracking. Unlike CCBP [29], no subject-specific calibration is required. Furthermore, the integrated VAE-SQI module provides explicit quality control absent from [18,20,30,31], preventing the model from learning spurious patterns from corrupted segments.

It should be noted that all comparisons in Table 6 are conducted across heterogeneous experimental settings, including different datasets, subject counts, age distributions, measurement sites, and radar configurations. No publicly standardized evaluation benchmark currently exists for radar-based BP estimation, and each study reports results exclusively on its own dataset. Consequently, Table 6 is intended to contextualize MARU-MTL within the current landscape of radar-based BP estimation rather than to claim strict superiority. Among the compared chest-measurement studies, MARU-MTL was evaluated on the largest and most demographically diverse cohort. Achieving competitive estimation accuracy under these challenging conditions provides meaningful evidence of the framework’s robustness.

### 4.2. Ablation Study

A comprehensive ablation study was conducted to systematically quantify the contribution of each proposed component to the overall performance. Specifically, four key design choices were evaluated, including the Bi-Mamba module, the multi-task learning framework, the VAE-SQI, the multi-scale attention bottleneck. All ablation experiments were performed under the same training configuration to ensure a fair comparison.

#### 4.2.1. Effect of Bi-Mamba Module

**Figure 15:**
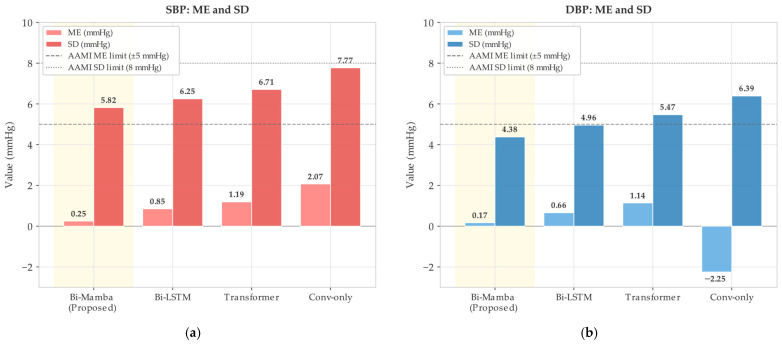
Comparison of ME and SD for different temporal modeling modules. (**a**) SBP estimation. (**b**) DBP estimation.

As shown in Figure 15, removing the Bi-Mamba module and relying solely on convolutional layers leads to a marked performance degradation. This result highlights the importance of explicit temporal modeling for blood pressure estimation from pulse-wave sequences.

Compared with Bi-LSTM, the Bi-Mamba module achieves lower ME for both SBP and DBP while also reducing computational overhead. Transformer-based modeling attains comparable accuracy; however, its quadratic complexity of *O*(*L*^2^) requires substantially more computational resources. In contrast, Bi-Mamba maintains linear complexity of *O*(*L*), offering a clear efficiency advantage, particularly when processing long RPW sequences.

#### 4.2.2. Effect of Multi-Task Learning

As shown in Table 7, the MTL framework significantly affects blood pressure estimation performance, and the loss weight λ determines whether the auxiliary task provides effective regularization. The best overall performance is obtained at λ = 0.01. In contrast, removing the MTL framework leads to a statistically significant performance drop, with SBP MAE increasing from 3.87 to 5.05 mmHg and the BHS grade declining from A to B. This difference was confirmed by a Mann–Whitney U test with a significance level below 0.001. However, BHS grades are discrete categorical thresholds, and therefore the 23.4% continuous reduction in MAE provides a more robust basis for evaluating MTL’s contribution than the grade transition alone. This result indicates that the auxiliary ABP waveform reconstruction task regularizes the model by constraining the shared encoder to learn richer hemodynamic representations, rather than exploiting superficial correlations.

When λ is increased beyond 0.01, the performance degrades progressively. This trend suggests that excessive emphasis on waveform reconstruction shifts representational learning away from the primary SBP/DBP regression objective. Consequently, morphological details that contribute limited information to scalar blood pressure estimation become more prominent, which weakens the extraction of BP-relevant features. Overall, the ABP reconstruction task is most effective when used as a lightweight regularization term, rather than being optimized with a weight comparable to the main task. This observation is consistent with the general principle of multi-task learning that auxiliary objectives should guide shared representation learning.

#### 4.2.3. Effect of VAE-SQI Screening

**Figure 16:**
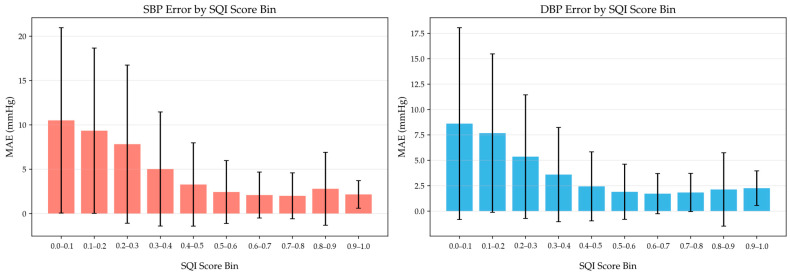
BP estimation error stratified by VAE-SQI quality score bins. Lower SQI scores correspond to poorer signal quality and substantially higher estimation error.

To evaluate the effectiveness of VAE-SQI, the relationship between blood pressure estimation error and the quality score was analyzed. Figure 16 presents the MAE for SBP and DBP across ten equally spaced SQI bins from 0.0 to 1.0, with SD shown as error bars. A clear association is observed between signal quality and estimation accuracy. Segments with SQI scores below 0.3 exhibit markedly larger errors, with the MAE for SBP exceeding 8 mmHg and the MAE for DBP exceeding 5 mmHg, accompanied by large SDs, indicating highly unreliable estimates. In contrast, for segments with SQI scores above 0.5, the MAE for SBP remains consistently below 3 mmHg, while the MAE for DBP stays below 2.5 mmHg, demonstrating substantially reduced errors.

It should be noted that the Pearson r of −0.316 between SQI and estimation error reflects only the linear component of their association. The relationship is markedly nonlinear, as evidenced by Figure 16: segments with SQI below 0.3 exhibit dramatically elevated errors, while those above 0.5 show consistently low errors. The Spearman rank correlation confirms a statistically significant monotonic association. Both correlation coefficients are moderate, which is expected since estimation error is determined by multiple factors beyond signal quality, including physiological state difficulty, inter-subject variability, and model limitations. Signal quality is a necessary but not sufficient condition for accurate estimation.

The strong correlation between SQI and estimation error confirms that VAE-SQI provides effective signal-quality screening. The sharp error increase in low-SQI bins indicates severe waveform corruption that fundamentally compromises blood pressure estimation, justifying the exclusion of these segments during both training and inference. Notably, the transition between the high-error and low-error regimes occurs around an SQI of approximately 0.5, offering empirical guidance for threshold selection. By filtering training and test data based on VAE-SQI scores, the proposed method suppresses low-quality inputs and improves the statistical reliability of blood pressure estimation.

To further quantify this effect, Table 8 compares the estimation performance evaluated on the filtered test set and the complete unfiltered test set.

As the retention ratio decreases from 100% to 70%, estimation accuracy improves consistently across all metrics, confirming that VAE-SQI effectively identifies and excludes corrupted segments. Although the 70% configuration yields slightly better performance than the selected 80% setting, we adopt 80% as the operating point for two reasons. First, retaining more data better preserves the representativeness of the test set and reduces the risk of reporting metrics on an overly curated subset, which directly addresses the concern of systematic optimism. Second, without filtering, the estimation errors fall outside the commonly referenced AAMI error thresholds, whereas the 80% setting achieves errors within the AAMI thresholds and metrics comparable to BHS Grade A with a moderate exclusion rate. The 80% retention ratio therefore represents a balanced trade-off between estimation accuracy and data coverage.

To justify the added complexity of VAE-SQI over simpler alternatives, Table 9 compares the quality discrimination metrics and downstream BP estimation performance under three screening strategies, all retaining 80% of test segments.

A clear performance hierarchy is observed. SNR-based screening provides minimal benefit, with an ROC-AUC of 0.394 below the random baseline and a weakly positive correlation with estimation error, indicating that spectral power ratio alone cannot capture RPW signal quality. This paradoxical result arises because hemodynamically provocative conditions produce stronger cardiac pulsations with higher SNR, yet these conditions are inherently the most challenging for BP estimation. The pre-screening score, combining three heuristic indicators via equal-weight fusion, achieves moderate improvement. VAE-SQI further reduces SBP MAE to 3.87 mmHg. This progressive improvement confirms that the learned latent representation in VAE-SQI captures quality-relevant patterns, justifying its added complexity.

#### 4.2.4. Effect of Multi-Scale Attention Bottleneck

As shown in Table 10, the contribution of each bottleneck component is evaluated by progressively incorporating the multi-scale design and the CTA attention mechanism. The baseline single-scale configuration without CTA yields the highest estimation errors. Introducing multi-scale parallel branches reduces both SBP and DBP errors, indicating that expanded receptive fields capture richer morphological patterns. The full multi-scale + CTA configuration achieves the best overall performance, confirming that attention-based feature recalibration and multi-scale receptive fields play complementary roles: the former enhances feature discriminability, while the latter ensures sufficient coverage of waveform patterns at different temporal scales.

### 4.3. Cross-Dataset Evaluation

To assess generalization across different data sources, two cross-dataset experiments were conducted: training on the public dataset and testing on the in-house dataset, and vice versa. The results are presented in Table 11.

An asymmetric performance pattern is observed. The Public/In-house direction achieves SBP MAE of 5.25 mmHg with errors remaining within the AAMI thresholds, suggesting that the richer training diversity supports partial generalization to an unseen clinical cohort including hypertensive and elderly subjects. In contrast, the In-house/Public direction shows substantially larger errors, primarily because the in-house training set provides fewer subjects and conditions, and the model encounters two unseen physiological states during testing. Cross-dataset generalization in this context constitutes a domain adaptation problem that lies beyond the scope of the current framework. Future work will explore transfer learning and domain adaptation strategies to improve cross-device and cross-population robustness.

### 4.4. Limitations and Future Work

Despite the promising results, this study has several limitations. The dataset, although the largest among comparable radar-based chest-measurement studies, remains relatively modest in scale compared with large-scale clinical databases. While the two datasets collectively span ages 21–75 years and include both normotensive and hypertensive individuals, the sample size may be insufficient to fully represent the physiological variability of the general population. To mitigate overfitting risk, data augmentation, regularization, and VAE-SQI screening are employed, and the cross-dataset evaluation provides preliminary evidence of generalizability; nevertheless, validation on larger and more heterogeneous cohorts is essential for clinical translation. To this end, a large-scale multi-center data collection effort targeting over 200 subjects with broader demographic and clinical coverage is currently underway in our group, which will provide a more rigorous basis for evaluating generalizability in future work.

The generalizability of the proposed method to broader populations, including individuals with diverse cardiovascular comorbidities, varying body mass index (BMI) levels, and different ethnic backgrounds, requires further validation. Furthermore, the main experiments were conducted with the radar positioned directly in front of the subject’s chest. A systematic multi-angle evaluation across the full datasets is planned for future work. In addition, subjects in the in-house dataset remained largely stationary during data acquisition. In real-world deployment scenarios, body movement, postural changes, and environmental interference may introduce additional signal degradation beyond the conditions covered by the current dataset.

In addition, the aggregate Pearson *r* reported in this study is largely influenced by inter-subject BP variability, as confirmed by the within-subject analysis in Section 3.3. Future evaluation should prioritize subject-stratified metrics such as per-subject MAE and within-subject correlation to provide a more rigorous assessment of continuous tracking performance.

Future work will focus on several directions. First, we plan to expand the dataset by recruiting larger and more diverse datasets to strengthen generalizability. Second, we will incorporate additional physiological modalities derived from the same radar sensor, such as heart rate variability and respiratory patterns. These signals may provide complementary hemodynamic cues and improve estimation accuracy. Third, lightweight model compression techniques will be explored to enable real-time deployment on edge devices for continuous bedside or home monitoring. Fourth, as demonstrated by the cross-dataset evaluation in Section 4.3, substantial performance degradation occurs when the model is transferred across datasets with different test environments and experimental protocols. Domain adaptation and transfer learning strategies, such as distribution alignment and adversarial training, will be investigated to bridge these distribution gaps and improve cross-dataset robustness without extensive retraining. Finally, the term “continuous” in this work refers to cuffless, unobtrusive, and persistently deployable monitoring, consistent with the established convention in the radar-based BP estimation literature, rather than implying beat-to-beat temporal resolution. The current framework estimates one BP value per 10 s interval, which is appropriate for hemodynamic trend monitoring but does not constitute true single-cycle estimation. Achieving beat-to-beat BP estimation from radar signals requires further advances in per-cycle waveform extraction robustness, real-time motion artifact suppression, and cycle-level model generalization. This represents an important direction that our research group is actively pursuing, and future work will explore shorter estimation windows and per-cycle inference strategies as radar signal processing techniques continue to mature.

## 5. Conclusions

This paper presents MARU-MTL, an end-to-end deep learning framework for continuous and contactless blood pressure estimation using millimeter-wave RPW. Three key contributions are made in the areas of signal quality assessment, network architecture design, and multi-task learning. First, a VAE-SQI mechanism is proposed to automatically assess and screen radar pulse wave quality in an unsupervised manner, providing unified quality control for both the training and inference stages without requiring manual annotation. Second, a Bidirectional Mamba module is integrated at the bottleneck of a U-Net backbone to efficiently model long-range temporal dependencies with linear computational complexity, overcoming the limitations of purely convolutional architectures while avoiding the quadratic cost of Transformer-based alternatives. Third, a multi-task learning strategy coupling BP regression with auxiliary ABP waveform reconstruction strengthens physiological consistency and provides effective implicit regularization.

Experiments on two synchronized radar–ABP datasets comprising 55 subjects across multiple physiological conditions demonstrate that MARU-MTL achieves state-of-the-art performance, with Pearson correlation coefficients of 0.944 and 0.937 for SBP and DBP, respectively. The method meets the error criteria specified by the AAMI standard and achieves the accuracy level corresponding to BHS Grade A for both SBP and DBP. Operating with a single radar sensor and requiring no additional contact-based devices or subject-specific calibration, the proposed framework offers a practical pathway toward continuous blood pressure monitoring in clinical and home settings.

## Figures and Tables

**Table 1:** Definition of VAE-SQI quality assessment components.

Component	Definition	Weight
Reconstruction consistency $S_{i}^{R}$	Measures conformity to VAE’s dominant mode based on reconstruction error $e_{i}=L^{-1}{\left\|{\tilde{x}}_{i}-{\hat{x}}_{i}\right\|}_{2}^{2}$	0.35
Latent space consistency $S_{i}^{L}$	Computes Euclidean distance from latent mean to high-quality center	0.15
Morphological features $S_{i}^{M}$	Quantifies RPW plausibility based on peak count, post-reconstruction peak preservation, and waveform smoothness	0.3
Physiological priors $S_{i}^{P}$	Directly adopts the pre-filtering score	0.2

**Table 2:** Performance metrics of VAE-SQI quality assessment.

Metric	Value
ROC-AUC	0.717
AP	0.89
F1-Score	0.864
Pearson *r*	−0.316 (*p* < 0.01)
Spearman ρ	−0.291 (*p* < 0.01)

**Table 3:** Radar parameter table.

Parameter	Value
Tx	1
Rx	4
Start frequency	77 GHz
Bandwidth	3 GHz
Frequency slope	50 MHz/μs
Idle time	15 μs
Ramp end time	60 μs
Sample points	64
Sample rate	2 MHz

**Table 4:** Overall blood pressure estimation performance of MARU-MTL.

Accuracy	SBP [95% CI]	DBP [95% CI]
ME (mmHg)	0.07 [−0.22, 0.36]	–0.03 [−0.27, 0.20]
SD (mmHg)	5.90 [5.43, 6.40]	4.61 [4.23, 5.02]
MAE (mmHg)	3.87 [3.64, 4.09]	2.93 [2.75, 3.11]
RMSE (mmHg)	5.90 [5.43, 6.41]	4.61 [4.23, 5.02]
*r*	0.944 [0.934, 0.953]	0.937 [0.925, 0.947]
≤5 mmHg (%)	75.7%	83.9%
≤10 mmHg (%)	92.1%	95.8%
≤15 mmHg (%)	97.0%	98.3%

CI: Confidence Interval.

**Table 5:** Per-dataset estimation performance.

Dataset	SBP (ME/SD/MAE/RMSE)	SBP *r*	DBP (ME/SD/MAE/RMSE)	DBP *r*	SBP/DBP BHS	SBP/DBP AAMI
Public (N = 30)	0.09/6.12/4.05/6.12	0.928	−0.05/4.82/3.08/4.82	0.924	A/A	Yes
In-house (N = 25)	0.25/6.55/4.42/6.55	0.901	−0.21/5.25/3.45/5.25	0.895	B/A	Yes
Pooled (N = 55)	0.07/5.90/3.87/5.90	0.944	−0.03/4.61/2.93/4.61	0.937	A/A	Yes

**Table 6:** Comprehensive performance comparison with state-of-the-art radar-based BP estimation methods.

Method	Measurement Site	Subject	SBP (ME/SD/MAE/RMSE)	SBP *r*	DBP (ME/SD/MAE/RMSE)	DBP *r*
CNN + LSTM [28]	Chest	30	2.04/12.65/9.30/NR	0.80	0.48/8.16/5.91/NR	0.85
TRCCBP [17]	Chest	31	−0.94/6.28/4.95/NR	NR	−0.62/6.46/4.53/NR	NR
CCBP [29]	Wrist	15	−1.30/6.17/4.42/NR	0.919	−3.10/4.93/4.33/NR	0.892
mmRBP [30]	Wrist	15	0.87/6.12/NR/6.00	0.88	0.59/3.78/NR/4.00	0.86
RSD-Net [18]	Chest	30	−0.32/6.14/4.61/6.14	0.84	−0.20/5.50/4.42/5.50	0.80
Two-stage model [20]	Chest	30	−1.09/5.15/5.00/6.24	0.933	−0.26/4.35/3.96/4.98	0.934
mmBP+ [31]	Wrist	33	0.65/3.92/NR/NR	NR	1.31/3.99/NR/NR	NR
DSFNN-BP [32]	Neck	40	0.49/5.19/4.16/NR	NR	−0.32/5.11/3.87/NR	NR
Ours	Chest	55	0.07/5.90/3.87/5.90	0.944	−0.03/4.61/2.93/4.61	0.937

Not Reported: NR.

**Table 7:** Ablation results for multi-task learning with different auxiliary loss weights λ.

Configuration	SBP MAE	SBP SD	SBP *r*	SBP BHS	DBP MAE	DBP SD	DBP *r*	DBP BHS	AAMI
without MTL	5.05	7.75	0.902	B	3.83	5.91	0.949	A	Yes
λ = 0.5	10.35	13.52	0.657	D	8.33	10.73	0.574	D	No
λ = 0.2	7.02	9.66	0.842	C	5.74	7.99	0.795	B	No
λ = 0.1	7.10	10.32	0.817	C	6.09	8.57	0.756	B	No
λ = 0.05	6.11	8.72	0.874	B	4.79	6.94	0.848	A	No
λ = 0.01 (Proposed)	3.87	5.90	0.944	A	2.93	4.61	0.937	A	Yes

**Table 8:** Effect of SQI filtering on test-set performance.

Configuration	SBP MAE	SBP SD	SBP *r*	SBP BHS	DBP MAE	DBP SD	DBP *r*	DBP BHS	AAMI
Without SQI filtering	5.33	7.61	0.856	B	4.57	6.92	0.851	B	No
With SQI filtering (90%)	4.31	6.69	0.891	B	3.64	5.39	0.883	A	Yes
With SQI filtering (80%)	3.87	5.90	0.944	A	2.93	4.61	0.937	A	Yes
With SQI filtering (70%)	3.68	5.69	0.948	A	2.81	4.57	0.939	A	Yes

**Table 9:** Comprehensive comparison of screening methods.

Method	ROC-AUC	AP	F1	Pearson r	Spearman ρ	SBP MAE	SBP MAE	SBP BHS	DBP MAE	DBP SD	DBP BHS	AAMI
SNR threshold	0.394	0.765	0.775	0.120	0.161	5.06	7.14	B	4.12	5.89	A	Yes
Pre-screening score	0.692	0.874	0.856	−0.292	−0.279	4.31	6.39	A	3.40	5.19	A	Yes
VAE-SQI (proposed)	0.717	0.890	0.864	−0.316	−0.291	3.87	5.90	A	2.93	4.61	A	Yes

**Table 10:** Ablation results for bottleneck design.

Configuration	SBP MAE	SBP SD	SBP *r*	SBP BHS	DBP MAE	DBP SD	DBP *r*	DBP BHS	AAMI
Single-scale without CTA	4.67	6.99	0.911	A	3.64	5.55	0.892	A	Yes
Multi-scale without CTA	4.26	6.47	0.920	A	3.18	4.90	0.917	A	Yes
Multi-scale + CTA	3.87	5.90	0.944	A	2.93	4.61	0.937	A	Yes

**Table 11:** Cross-dataset evaluation results.

Training Set/Test Set	SBP (ME/SD/MAE/RMSE)	SBP *r*	DBP (ME/SD/MAE/RMSE)	DBP *r*	SBP/DBP BHS	SBP/DBP AAMI
Public/In-house	0.85/7.12/5.25/7.17	0.865	0.42/5.65/4.18/5.67	0.852	B/B	Yes
In-house/Public	5.42/8.35/7.15/9.95	0.784	3.15/8.12/6.05/8.71	0.765	C/B	No
Pooled (proposed)	0.07/5.90/3.87/5.90	0.944	−0.03/4.61/2.93/4.61	0.937	A/A	Yes

## Data Availability

The public dataset used in this study is available as described in [25]. The newly collected synchronized radar–ABP dataset is not publicly available due to privacy/ethical restrictions, but it is available from the corresponding author upon reasonable request.
